# Tobacco Exposure and Complications in Conservative Laryngeal Surgery

**DOI:** 10.3390/cancers6031727

**Published:** 2014-08-19

**Authors:** Francesca Romana Fiorini, Alberto Deganello, Guglielmo Larotonda, Giuditta Mannelli, Oreste Gallo

**Affiliations:** Academic Clinic of Otolaryngology-Head and Neck Surgery, University of Florence, Via Largo Brambilla 3, 50134 Florence, Italy; E-Mails: adeganello@hotmail.com (A.D.); guglielmolaro@libero.it (G.L.); mannelli.giuditta@gmail.com (G.M.); o.gallo@med.unifi.it (O.G.)

**Keywords:** head and neck cancer, postoperative complications, smoking status

## Abstract

Smoking is an important risk factor in the development of head and neck cancer. However, little is known about its effects on postoperative complications in head and neck cancer surgery. We performed a retrospective analysis on 535 consecutive laryngeal cancer patients submitted to open partial laryngectomy at the Otolaryngology-Head and Neck Surgery Department of Florence University to evaluate a possible correlation between smoking and surgical complications. Patients were grouped in non smokers and smokers and evaluated for airway, swallowing, local and fistula complications by multivariate analysis: 507 (95%) patients were smokers, 69% presented supraglottic, 30% glottic and 1% transglottic cancer. The most common operation was supraglottic horizontal laryngectomy in 58%, followed by supracricoid partial laryngectomy in 27% and frontolateral hemilaryngectomy in 15% of cases. The incidence of overall complications was 30%, airway complications representing the most frequent (14%), followed by swallowing (7%), local (6%) and fistula complications (3%). Smokers developed more local complications (*p* = 0.05, univariate, *p* = 0.04, multivariate analysis) and pharyngocutaneous fistula (*p* = 0.01, univariate, *p* = 0.03, multivariate analysis).

## 1. Introduction

Smoking is the most important risk factor in head and neck cancer [1]. According to a large case-control study, over 90% of laryngeal cancer cases in Southern Europe could be prevented by avoiding smoking [2]. Its role in the carcinogenetic process, the mechanisms of DNA genetic changes, mutations in oncogenes and tumor suppressor genes have already been described in lung and head and neck cancer [3,4,5]. Free radicals and oxidative damage in tobacco-related carcinogenesis have also been investigated in inhalation studies of tobacco smoke in laboratory animals [6,7,8,9,10].

However, tobacco exposure also represents a risk factor for the development of perioperative surgical complications [11]. Robbins *et al*. [12] described impaired nutritional status and alcohol consumption, duration of surgery, type of surgical wound, complexity of the procedure, use of flaps, blood replacement and the use of drains, nasogastric tubes and tracheostomies as the major causes of wound infections. Moreover, head and neck cancer treatment often includes a combination of surgery, radiotherapy and chemotherapy, which increase the difficulty of wound healing, especially in a population with a mean age of 60 years, with a higher incidence of comorbidities and a preoperative debilitated physical state [13,14]. Smokers with head and neck cancer have considerable difficulty tolerating these treatments, due to smoking-related comorbidities such as vascular, cardiac and pulmonary disease.

Wound healing is fundamental in a region where function and anatomic form are strictly interdependent. Head and neck surgical procedures require entry into the mucosa of the aerodigestive tract, exposing the surgical wound to oropharyngeal secretions and bacteria [15,16]. Multiple factors contribute to poor wound healing. In head and neck cancer surgery this can result in exposure of vital structures, including the great vessels [17,18], infection and pharyngocutaneous fistula, and the potential for life-threatening bleeding.

While the relationship between smoke and wound healing has been studied for reconstructive head and neck cancer surgery [19,20], little is known about non-reconstructive surgery. Hence, we focused on a series of consecutive patients submitted to conservative laryngeal surgery, in order to analyze the impact of smoke on wound healing in this specific subgroup, and define possible risk factors responsible for the onset of postoperative complications.

## 2. Experimental

We performed a retrospective analysis on 535 consecutive patients submitted to open partial laryngectomy for primary or recurrent laryngeal squamous cell carcinoma between 1982 and 2007, treated at the Otolaryngology-Head and Neck Surgery Department of Florence University.

Table 1 shows patient and tumor characteristics. Ages ranged from 24 to 79 years (mean 57.9, median 59); the majority (95%) of patients were smokers and about half of them (48%) were also alcohol abusers.

Early stage tumors (classified as T1 and T2) were the most common ones (almost 90%). In 69% of cases the region involved was the supraglottic larynx, in 20% of patients we found positive neck lymph nodes at clinical examination.

Table 2 gives details of surgery for patients submitted to primary partial laryngectomy (PPL) and salvage partial laryngectomy (SPL) following radiotherapy.

**Table 1:** Patient and tumor characteristics.

Characteristics	Aspects	Number of Patients (535)
Age group	<60 years	285 (53%)
	≥ 60 years	250 (47%)
Smoking status	Non smokers	28 (5%)
	Smokers	507 (95%)
Alcohol consumption	No alcohol consumption	281 (52%)
	Alcohol abuse	254 (48%)
Tumor subsite	Glottic tumor	154 (30%)
	Supraglottic tumor	372 (69%)
	Transglottic tumor	9 (1%)
cT * classification	1	251 (48%)
	2	227 (42%)
	3	41 (7%)
	4	16 (3%)
cN ** classification	0	433 (80%)
	1	73 (14%)
	2	27 (5.6%)
	3	2 (0.4%)

* cT = clinical tumor stage, ** cN = clinical neck stage.

**Table 2:** Details of laryngectomy.

Features	Aspects	Number of Patients		
		All patients (*n* = 535)	Primary PL * (*n* = 516)	Salvage PL * (*n* = 19)
Type	Frontolateral	82 (15%)	82 (16%)	0 (0%)
	CHP **	105 (20%)	92 (18%)	13 (68%)
	CHEP ***	39 (7%)	33 (6%)	6 (32%)
	SHL ʃ	309 (58%)	309 (60%)	0 (0)
Neck dissection	No	282 (53%)	265 (51%)	17 (89%)
	Yes	253 (47%)	251 (49%)	2 (11%)
Type of neck dissection	None	282 (53%)	265 (51%)	17 (90%)
	SND ʃ ʃ	48 (9%)	47 (9%)	1 (5%)
	mRND ʃ ʃ ʃ	205 (38%)	204 (40%)	1 (5%)

* PL = partial laryngectomy, ** CHP = crico-hyoido-pexy, *** CHEP = crico-hyoid-epiglotto-pexy, ʃ SHL = supraglottic horizontal laryngectomy, ʃ ʃ SND = selective neck dissection, ʃ ʃ ʃ mRND = modified radical neck dissection.

Five hundred and sixteen (95%) patients had PPL, while 19 patients (3.5%) had SPL. Supraglottic horizontal laryngectomies (SHL) was the most frequently performed operation (58%), followed by supracricoid partial laryngectomies (27%), including both cricohyoidopexy (CHP) or cricohyoido-epiglottopexy (CHEP), and frontolateral laryngectomies (FLHL) in 15% of cases. Two hundred and fifty-three patients (47%) were also submitted to neck dissection.

According to our Institutional policy, all patients eligible for partial surgery showed at preoperative evaluation: Karnofsky physical index superior to 80%, no dyspnea whilst climbing two flights of stairs, satisfactory pulmonary functional tests according to Morris and Koski [21] and basal pO2 values above 85 mmHg. Furthermore, patients with severe cardio-vascular diseases, diabetes mellitus, gastro-oesophageal reflux, severe chronic obstructive pulmonary disease and rheumatoid arthritis were considered not eligible for partial laryngectomy. Follow-up times ranged from 2 to 372 months (mean 74.6 months, median 60.5 months).

In agreement to the existing literature [22] complications were categorized into local (surgical wound infection or dehiscence, subcutaneous emphysema, operative site bleeding, dehiscence of pexy), swallowing (hypopharyngeal/laryngeal stenosis and dysphagia), airway (broncho-tracheal stenosis and pneumonia) and fistula. To identify patient and tumor factors predictive of complications we performed univariate analysis according to Fisher’s exact test, considering age, smoking and alcohol status, T classification, N classification, primary radiation treatment and type of partial laryngectomy as dependent variables. Logistic regression multivariate analysis was performed entering significant data at the univariate analysis. A *p* value less or equal to 0.05 was considered statistically significant. Statistical analysis was conducted with STATA 9.1 (Stata Corporation, College Station, TX, USA).

## 3. Results and Discussion

Table 3 summarizes the proportion of patients with postoperative complications. Overall, 30% of patients developed a postoperative complication following partial laryngectomy. Airway complications were the most frequent ones as they occurred in 73 patients (14%); swallowing complications occurred in 39 patients (7%); local complications and laryngocutaneous fistula had lower incidence rates, having affected 33 patients (6%) and 15 patients (3%), respectively.

**Table 3:** Postoperative complications after partial laryngectomy.

Complication		Number of Patients			
		All Patients (*n* = 535)	Primary PL * (*n* = 516)	Salvage PL * (*n* = 19)	*p* value
**Overall**	No	375 (70%)	359 (70%)	16 (84%)	0.61
	Yes	160 (30%)	157 (30%)	3 (16%)	
**Local**	No	502 (94%)	483 (94%)	19 (100%)	1.0
	Yes	33 (6%)	33 (6%)	0 (0%)	
**Swallowing**	No	496 (93%)	479 (93%)	17 (89%)	0.68
	Yes	39 (7%)	37 (7%)	2 (11%)	
**Airway**	No	462 (86%)	443 (86%)	19 (100%)	0.34
	Yes	73 (14%)	73 (14%)	0 (0%)	
**Fistula**	No	520 (97%)	502 (97%)	18 (95%)	0.48
	Yes	15 (3%)	14 (3%)	1 (5%)	

* PL = partial laryngectomy.

There were no statistically significant differences concerning the incidence of overall complications between patients having primary partial laryngectomy and patients having salvage partial laryngectomy following radiotherapy failure (*p* = 0.11).

In Table 4, we examine factors that are potentially related to the incidence of local complications. Smoking status (*p* = 0.05) and alcohol abuse (*p* = 0.03) were statistically associated to a major incidence of local complications upon univariate analysis. Smoking and alcohol abuse resulted statistically significant upon multivariate analysis (*p* = 0.04 and *p* = 0.03, respectively).

For fistula complications (Table 5), smoking status resulted statistically significant upon univariate (*p* = 0.01) and multivariate analysis (*p* = 0.03). Fistula did not seem to be related to prior irradiation. We did not observe any other correlation between smoke habit and systemic complications such as dysphagia and airway complications.

**Table 4:** Factors predictive for local complications.

Characteristics	Aspects	Patients (*n* = 535)	Complicated	Univariate	Multivariate
				*p* value	*p* value
**Age**	<60 years	285 (53%)	19 (7%)	0.719	0.845
	>=60 years	250 (47%)	14 (6%)		
**Smoking status**	Non smokers	28 (5%)	1 (3.57%)	0.05	0.04
	Smokers	507 (95%)	32 (6.3%)		
**Alcohol consumption**	No alcohol	281 (52%)	11 (4%)	0.03	0.03
	Alcohol abuse	254 (48%)	22 (9%)		
**Tumor Site**	Supraglottic	372 (69%)	27 (7%)	0.123	0.09
	Glottic	154 (30%)	6 (4%)		
	Transglottic	9 (1%)	0 (0%)		
cT *	1	253 (48%)	13 (5%)	0.705	0.962
	2	226 (42%)	16 (7%)		
	3	40 (7%)	3 (7%)		
	4	16 (3%)	1 (6%)		
cN **	0	433 (80%)	24 (6%)	0.078	0.160
	1	73 (14%)	5 (7%)		
	2	27 (5.6%)	3 (10%)		
	3	2 (0.4)	1 (50%)		
**Surgery type**	Frontolateral	82 (15%)	2 (2%)	0.277	0.069
	CHP ʃ	106 (20%)	9 (9%)		
	CHEP ʃ ʃ	38 (7%)	4 (11%)		
	SHL ʃ ʃ ʃ	309 (58%)	18 (6%)		
Previous RT ***	No	516 (96%)	33 (6%)	1.000	0.981
	Yes	19 (4%)	0 (4%)		

* cT = clinical tumor stage, ** cN = clinical neck stage, *** RT = radiotherapy, ʃ CHP = crico-hyoido-pexy, ʃ ʃ CHEP = crico-hyoid-epiglotto-pexy, ʃ ʃ ʃ SHL = supraglottic horizontal laryngectomy.

**Table 5:** Factors predictive for pharyngocutaneous fistula.

Characteristics	Aspects	Patients (*n* = 535)	Complicated	Univariate	Multivariate
				*p* value	*p* value
**Age**	<60 years	285 (53%)	9 (3%)	0.794	0.711
	>=60 years	250 (47%)	6 (2%)		
**Smoking status**	Non smokers	28 (5%)	0 (0%)	0.01	0.03
	Smokers	507 (95%)	15 (2.96%)		
**Alcohol consumption**	No alcohol	281 (52%)	5 (2%)	0.189	0.124
	Alcohol abuse	254 (48%)	10 (4%)		
**Tumor Site**	Supraglottic	372 (69%)	12 (3%)	0.570	0.132
	Glottic	163 (30%)	3 (2%)		
cT *	Transglottic	9 (1%)	0 (0%)		
	1	253 (48%)	6 (2%)	0.901	0.612
	2	226 (42%)	8 (4%)		
	3	40 (7%)	1 (2%)		
	4	16 (3%)	0 (0%)		
cN **	0	433 (80%)	13 (3%)	0.075	0.923
	1	73 (14%)	1 (1%)		
	2	27 (5.6%)	0 (0%)		
	3	2 (0.4%)	1 (50%)		
**Surgery type**	Frontolateral	82 (15%)	1 (1%)	0.556	0.319
	CHP ʃ	106 (20%)	4 (4%)		
	CHEP ʃ ʃ	38 (7%)	2 (5%)		
	SHL ʃ ʃ ʃ	309 (58%)	8 (3%)		
Previous RT ***	No	516 (96%)	14 (3%)	0.351	0.519
	Yes	19 (4%)	1 (5%)		

* cT = clinical tumor stage, ** cN = clinical neck stage, *** RT = radiotherapy, ʃ CHP = crico-hyoido-pexy, ʃ ʃ CHEP = crico-hyoid-epiglotto-pexy, ʃ ʃ ʃ SHL = supraglottic horizontal laryngectomy.

Smoking is the most important risk factor in head and neck cancer [1]. In this series 95% of patients were smokers. Supraglottic cancer developed in about 70% of cases. Only 3% of patients were submitted to open partial laryngectomy for a recurrence after radiotherapy failure while the remaining 97% were primary partial laryngectomy. The incidence of overall complications was 30%, airway complications representing the most common (14%), followed by swallowing (7%), local (6%) and pharyngocutaneous fistula complications (3%). Smoking status and alcohol abuse were statistically associated to a major incidence of local complications upon univariate analysis (*p* = 0.05 and *p* = 0.03, respectively). Also upon multivariate analysis, smoking and alcohol abuse resulted statistically significant (*p* = 0.04 and *p* = 0.03, respectively). Smoking status was also associated to an increased incidence of pharyngocutaneous fistula complications upon univariate (*p* = 0.01) and multivariate analysis (*p* = 0.03).

Although most head and neck surgeons believe that smoking causes increased postsurgical complications, there is lack of definitive data supporting this hypothesis. Recently, Lassig *et al.* [23] reviewed the existing evidence on this topic, examining the potential effects of tobacco on perioperative complications. Conclusions were relatively consistent among the 36 studies reviewed: approximately half of them showed the association between tobacco and perioperative complications. However, only the study of Marin *et al.* [24] classified tobacco use in an objective well-described manner using biomarkers and a tobacco questionnaire. In his series, of the 89 patients submitted to 101 pedicled and/or microvascular free flaps, about 37% suffered from minor wound healing or major complications. The relative risk for a complication was approximately 2-fold for those patients with an elevated preoperative serum cotinine concentration (*p* = 0.028). Wound complication rate was higher with a serum cotinine concentration greater than 10 ng/mL, but the risk had a trend to the statistical significance only among females (*p* = 0.054). He concluded that serum cotinine concentration might accurately predict the risk of wound complications after head and neck flap reconstruction.

The literature that highlights the negative impact of smoking is clearer regarding systemic complications as well as in microvascular reconstructive surgery after head and neck cancer extirpation [19,20,24]. In our study, the smoking status showed a significant impact only on the development of local and fistula complications, while airway and swallowing ones were not significantly increased in smokers. These findings have been previously reported by Gallo *et al.* [25] for the risk of postoperative pneumonia.

It is unclear how tobacco exposure may negatively influence the wound healing process. Of course in smoking patients the incidence of comorbidities is very high and the vascular bed is strongly altered. Vascular damage may delay wound healing, therefore smokers who undergo a surgical procedure may have a greater risk.

The mechanisms of vascular damage from smoking are well known [26]. Absorbed nicotine stimulates the release of catecholamines, whilst other products injure the arterial endothelium and promote atherogenesis. Free radicals and aromatic compounds diminish the endothelial synthesis of nitric oxide, causing impaired endothelium-dependent relaxation of arteries. The increased oxidation of low density lipoprotein (LDL) in smokers has synergistic effects in promoting monocyte adhesion and monocyte migration in the subintimal space. Continued stimulation of intimal cells by oxidized LDL leads to the development of atherosclerosis. Smoking also potentiates thrombosis at the dysfunctional endothelium by increasing the concentration of plasma fibrinogen and altering the activity of platelets. All these proatherogenic effects of smoking are also observed, even if to a lesser extent, in passive smokers. Ejaz *et al.* [27] using a well-defined chicken dorsum excision wound assay, found that different components of sidestream whole smoke solutions may have a cumulative negative impact on wound healing and related angiogenesis.

Previous clinical trials on the association between alcohol consumption and postoperative complications reported conflicting results. A recent systematic review and meta-analysis [28] evidenced how alcohol drinking is not an independent risk factor for surgical site infection and anastomotic leakage in gastrointestinal surgery. To our knowledge there exists no study which addresses the same issue in the field of otorhinolaryngology. Herein, we were the first to report an association between alcohol abuse and local complications.

In summary, we documented a clear significantly statistical association between smoking exposure and postoperative complications in conservative laryngeal cancer surgery. Of course, our preliminary data need to be supported by future further prospective trials in which tobacco exposure is better analyzed from the biochemical point of view.

## 4. Conclusions

Tobacco exposure is implicated not only in the pathogenesis of head and neck cancer but also seems to play a key role in the development of local complications in patients who undergo conservative laryngeal cancer surgery.
